# Preliminary Studies on the In Vitro Interactions Between the Secondary Metabolites Produced by Esca-Associated Fungi and Enological *Saccharomyces cerevisiae* Strains

**DOI:** 10.3390/plants11172277

**Published:** 2022-08-31

**Authors:** Leonardo Scarano, Francesco Mazzone, Francesco Mannerucci, Margherita D’Amico, Giovanni Luigi Bruno, Antonio Domenico Marsico

**Affiliations:** 1 Department of Soil, Plant and Food Sciences (Di.S.S.P.A.), University of Bari Aldo Moro, Via G. Amendola, 165/A, 70126 Bari, Italy; 2 Council for Agricultural Research and Economics—Research Centre for Viticulture and Enology (CREA-VE), Via Casamassima, 148, 70010 Turi, Italy

**Keywords:** pullulan, pentaketides naphthalenone, scytalone, isosclerone, Esca complex diseases, microvinification, growth, enological performance

## Abstract

Esca-affected vines alter the carbohydrate metabolism, xylem transport of water and photosynthesis and show regular grapes (but berries do not reach maturity), and phenolic compounds are reduced in concentration, oxidate and polymerizate. Pullulan and a mixture of scytalone and isosclerone (9:1; *w*/*w*), secondary metabolites produced in vitro and in planta by *Phaeoacremonium minimum* (syn. *P. aleophilum*) and *Phaeomoniella chlamydospora,* were assayed against the strains Byosal HS1 and IOC 18-2007 in microvinifications with synthetic grape must. The presence of pullulan and pentaketides mix affects the growth and metabolism of the tested *Saccharomyces cerevisiae* strains. Assays at 100 and 1000 µg mL^−1^ inhibited the growth of both strains, while no effects were recorded when evaluated at 1 and 5 µg mL^−1^. In comparison with the controls, pullulan and the scytalone/isosclerone mixture at 10 µg mL^−1^ had a growth reduction, a lower alcohol yield, reduced the concentration of tartaric acid and malic acid; and slowed down the production of lactic acid, acetic acid and total polyphenol content of the tested *S. cerevisiae* strains. These metabolites could be applied as an alternative to the sulfite addition in the early stages of vinification to support the action of selected *Saccharomyces*. Appealing is the subtractive action of pullulan against tartaric acid. Further data are needed to confirm and validate the enological performance in freshly pressed grape juice.

## 1. Introduction

Grapevine (*Vitis vinifera* L.) cultivation and winemaking production are negatively affected by the impacts of pathogens and pests on vines, which compromise their annual growth cycle, lifetime and organoleptic qualities of grapes and wine. Grapevine trunk diseases (GTDs) are the most destructive diseases of a vineyard, causing cankers, dieback and the eventual death of vines. The presence of a consortium of fungi participates in GTDs. Species of *Botryosphaeria*, *Diplodia*, *Dothiorella*, *Granulodiplodia*, *Lasiodiplodia*, *Neofusicoccum*, *Phaeobotryosphaeria*, *Spencermartinsia*, *Phaeomoniella*, *Cadophora*, *Phaeoacremonium*, *Fomitiporia*, *Fomitiporella*, *Fuscoporia*, *Inocutis*, *Phellinus*, *Porodaedalea*, *Stereum*, *Eutypa*, *Eutypella*, *Cryptosphaeria*, *Cryptovalsa*, *Diatrype*, *Diatrypella*, *Pleurostomophora* and *Diaporthe* are isolated from grapevine wood affected by GTDs. Fungi in the Diaporthales order are implicated in Esca and other GTDs, such as the Eutypa dieback [1,2,3,4,5].

The disease usually called “Esca” is one of the longest recognized and most complex and destructive GTDs [2]. In particular, the worldwide cost for the replacement of dead vines is roughly estimated to be more than 1.0 billion euros per year [3]. 

Esca is now considered a complex of different diseases overlapping in the same vine or developing at different ages of the vine’s life span. Esca complex includes white rot (which is at the origin of the name Esca) and three vascular syndromes: brown wood streaking of grapevine cuttings, Petri disease and grapevine leaf stripe disease (GLSD, previously named “young esca”). GLSD affects young and old vines, which show wood streaking and discoloration. The association between GLSD and white rot was described as “Esca proper”. The vines affected by Esca complex, white rot and Esca proper can show apoplectic symptoms. Brown wood streaking, Petri disease and GLSD are grouped as phaeotracheomycosis complex [2,6,7,8]. Symptoms on leaves start with chlorotic areas becoming necrotic and conferring a “tiger striped” appearance. On berries, dark purple spots on the epidermis can be observed [9,10]. The manifestation of leaf and berry symptoms and apoplexy may fluctuate from year to year [1,2,4,6,7,8,9].

Members of the basidiomycetous genus *Fomitiporia* (*F. mediterranea* M. Fisch. mainly in Europe and the Mediterranean area) cause white rot [11,12]. *Phaeomoniella chlamydospora* (W. Gams, Crous, M.J. Wingf. & Mugnai) Crous & W. Gams, *Phaeoacremonium minimum* (Tul. & C. Tul.) Gramaje, L. Mostert & Crous (syn. *Phaeoacremonium aleophilum* W. Gams, Crous, M.J. Wingf. & Mugnai) and other *Phaeoacremonium* ssp. are the etiological agents of brown wood streaking, Petri disease and GLSD [1,7].

So far, no curative methods are available. Different strategies are applied to limit the occurrence of GTDs, both in nurseries and fields [2]. Most promising is the application of biocontrol agents. Strains of *Pythium oligandrum* Drechsler, bacteria of the genus *Paenibacillus* and mycoparasite fungi of the genus *Trichoderma* spp. (including *Trichoderma atroviride* P. Karst., *Trichoderma asperellum* Samuels, Lieckf. & Nirenberg and *Trichoderma gamsii* Samuels & Druzhin.), are applied against GTDs, including Esca [2,10,13], still with innovative, ecofriendly hybrid nanomaterials [13].

No pathogens have been isolated from the leaves or berries of infected plants. Leaf “tiger stripe” and berry symptoms are linked to cultivar susceptibility, vine age, the microorganisms involved, pedoclimatic conditions and other physiological factors [6,7,8]. Reaction products of the diseased wood, stress metabolites, oxygen radicals, polyphenol compound changes, phytotoxic metabolites excreted by Esca-associated fungi, or a combination of these, originating in the discolored woody tissues of affected trunks and branches contribute to the deterioration/necrosis in the wood and leaves symptoms development [2,4,5,6,8,10,14,15,16,17,18,19,20,21]. Glycerophospholipids and polyphenol compounds, principally stilbenoids could play a role to limit in planta Esca pathogens development [10]. Macro- and micronutrients play a role in Esca complex symptom progression [22,23,24]. 

**Figure 1:**
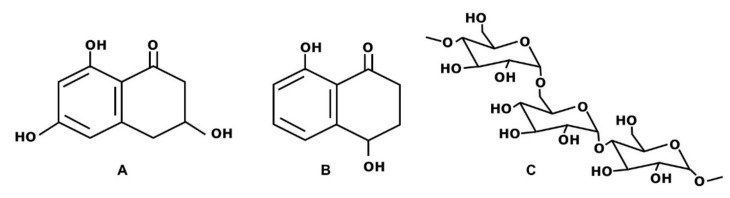
Structure formulae of scytalone (**A**), isosclerone (**B**) and pullulan (**C**).

*P. minimum* and *P. chlamydospora* produce different secondary metabolites in vitro: naphthalenone pentaketides, exopolysaccharides and polypeptides. Among naphthalenone pentaketides, from liquid culture of *P. minimum* were isolated scytalone, isosclerone (Figure 1), *cis*-4-hydroxy-scytalone, 1,3,8-trihydroxynaphtalene (1,3,8-THN), 2,4,8-trihydroxytetralone (2,4,8-THT), 3,4,8-trihydroxytetralone (3,4,8-THT), flavioline, 2-hydroxyjuglone (2-HJ) and 4-hydroxybenzaldehyde. From liquid cultures of *P. chlamydospora*, scytalone, isosclerone, 4-hydroxybenzaldehyde, tyrosol, 1-O-methylemodine, 3-hydroxy-5-decanolide, (S)-4-hydroxyphenyllactic acid and 4-hydroxy-3-(3-methyl-2-butenyloxy)-benzoic acid have been yielded. The exopolysaccharides include the α-glucan pullulan (Figure 1) [14,15,25,26,27,28,29]. The structures of the polypeptides have not yet been determined [15,29]. 

The biological activity of metabolites secreted by *P. minimum* and *P. chlamydospora* was evaluated against grapevine leaves, calli and protoplasts and towards *Arabidopsis thaliana* protoplasts [14,15,25,26,27,28]. Assayed on detached leaves of cvs Italia, Sangiovese and Matilde, aqueous solutions of scytalone (50 µg mL^−1^) produce light green to chlorotic, round to irregular, interveinal or marginal spots, which eventually coalesce or diffuse to large areas. Similarly, isosclerone (100 µg mL^−1^ in water) origins yellowish spots, slowly becoming coalescent and necrotic, followed by distortion and withering of the lamina [25,26]. Pullulan (50 µg mL^−1^) causes the collapse of marginal and interveinal tissue, which desiccates and becomes dry [27]. 

Polypeptides secreted by *P. chlamydospora* and *P minimum* stimulate anthocyanins production on grapevine leaves. Applied to grapevine cells in culture, these polypeptides have modified proton fluxes, depolarized the cell membrane, inhibited the transport of sucrose and glutamine and, lastly, caused the death of the cells [15,29].

Variations in glutathione/ascorbate pools, PR-proteins, phenolic compounds, phytoalexins, carbohydrate metabolism, xylem water transport and photosynthesis are observed in Esca complex-affected vines [4,8,10,14,15,16,17,18,19,20,21,30,31,32,33,34,35,36]. Climatic conditions also influence vine physiology, pathogens aggressiveness, their interaction, tiger stripes and apoplexy expression [37,38].

Vines with Esca complex show regular bunches with grapes that do not reach maturity, loss of grape quality and yield, variations in the concentration of flavonoids, anthocyanins, phenolic compounds and their polymerization [17,22,39]. *Trans*-resveratrol concentration increases in wood with symptoms of brown streaks, in the berries of vines infected with *P. minimum* and *P. chlamydospora* [14,17,28] and in Trebbiano d’Abruzzo wines obtained from Esca complex diseased grapevine [22,39].

Laccases produced by *F. mediterranea* could oxidize proanthocyanidins and anthocyanins, contributing to the reduction of phenolic compounds concentration in wines obtained from Esca complex diseased vines [40]. Anthocyanins and proanthocyanidins, present in the grape skins and seeds, are phenolic compounds involved in the quality of red wine. Anthocyanins are responsible for the color of red wine [41], while proanthocyanidins include different phenolic compounds comprised of flavan-3-ol monomer subunits and confer astringent and bitter properties [42] and play a role in long-term color stability [43,44]. In addition, musts obtained from Esca complex diseased vines show a reduction in sugars (up to 4%) with an increase in total acidity and assimilable nitrogen contents associated with a delayed ripening of berries and reduced photosynthesis [36,39,40].

In winemaking production, pure cultures of selected strains of yeast, sourced in the house or supplied from specific producing companies, are commonly used as starter cultures. The current study aimed to characterize the incidence of exopolysaccharide pullulan and the two pentaketides produced by *P. minimum* and *P. chlamydospora* on growth and enological parameters of *Saccharomyces cerevisiae* (Desm.) Meyen strains of enological interest using a synthetic must as substrate.

## 2. Results

### 2.1. Pullulan and Pentaketides Production, Extraction and Purification

Pc25 and Pm33 grown in CzMM as stationary liquid cultures revealed differences in growth rate, final pH and secondary metabolites production (Table 1).

### 2.2. Pullulan and Pentaketides Mix Interaction with Saccharomyces Cerevisiae Strains

**Figure 2:**
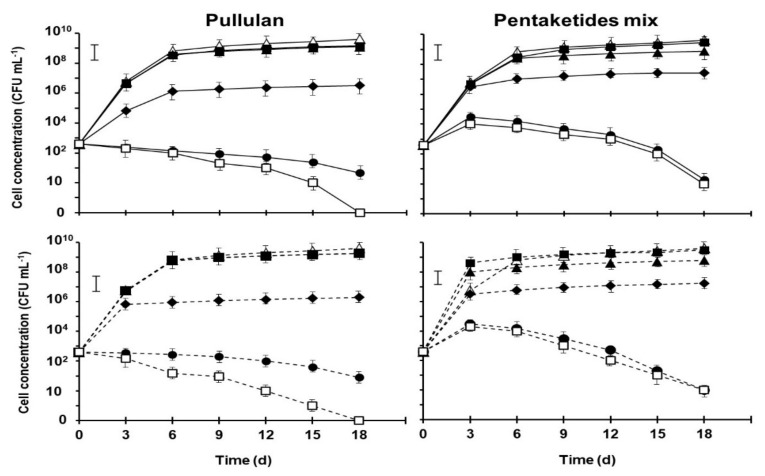
Growth curves of *Saccharomyces cerevisiae* strains Byosal HS1 (—) and IOC 18-2007 (---) growth on synthetic must containing 0 (△), 1 (■), 5 (▲), 10 (◆), 100 (•) or 1000 (□) µg mL^−1^ of pullulan or pentaketides mix (scytalone/isosclerone; 9:1/*w*/*w*) during 18 days of fermentation (20 ± 1 °C, 12 rpm orbital shaking, in the dark). Values are the means of 2 experiments with 3 replicates ± sd. The vertical bars show the LSD value (*p* = 0.05). CFU = colony-forming units.

On synthetic must (Figure 2), strain Byosal HS1 reached 4.1 × 10^9^ CFU mL^−1^, while 3.8 × 10^9^ CFU mL^−1^ were counted for IOC 18-2007. Increasing concentrations of pullulan or pentaketides mix reduced the growth and viability of the two isolates until complete inhibition at 100 and 1000 µg mL^−1^.

Based on the results of growth experiments, the concentration of 10 µg mL^−1^ was selected to verify the effects of for both pullulan and pentaketides on the enological performance of Byosal HS1 and IOC 18-2007 (Table 2).

The pH, from the initial 3.4, was reduced in all the cultures considered (Table 2). 

Utilized sugars (Table 2) were reduced in the presence of pentaketides mix. Pullulan did not affect sugar utilization from both tested strains.

The presence of pullulan and pentaketides mix reduced the percentage of ethanol produced (Table 2).

The ethanol yield (Table 2) was not significantly different from the two strains grown in the controls. Compared to control, strain IOC 18-2007 showed an insignificant higher overall when grown with pentaketides mix.

The presence of pullulan reduced total acidity (Table 2). 

The volatile acidity (Table 2) was related to the substrate and the strain considered. Byosal HS1 grown in the presence of pullulan produced the lowest volatile acidity.

The initial content of tartaric acid (5 g L^−1^) was reduced especially in the media containing pullulan (Table 2).

Both strains utilized more than 50% of the malic acid present in the synthetic must (Table 2).

Lactic acid was produced by both strains (Table 2). Pullulan allowed the lower accumulation.

In the media performed as control fermented by strain Byosal HS1, the concentrations in total polyphenols were almost two times those of strain IOC 18-2007. The presence of pullulan and pentaketides mix almost halved the content of these substances in fermentations conducted with the Byosal HS1 strain in comparison with the control (Table 2). 

Pullulan and pentaketides mix lowered the fermentation vigour of both *S. cerevisiae* strains (Table 2).

## 3. Discussion

The alcoholic fermentation of grape must is a biological process allowing the transformation of grape juice into wine. During fermentation, sugars contained in the must, by the action of yeast’s enzymes, are transformed into ethanol, carbon dioxide and a series of by-products that will compose the aromatic and gustatory qualities of the wine. Spontaneous alcoholic fermentation is initiated by a complex yeast community comprising the enological microbiome of berries (e.g., species of *Torulopsis*, *Cryptococcus*, *Candida*, *Rhodotorula*, *Hanseniaspora*, *Kloeckera*, *Metschnikowia* and *Pichia*). These yeasts are involved during the first 3–4 days, after which they die off, due to the rapid depletion of nutrients and oxygen, and the toxicity of the accumulation of ethanol and other metabolites [45,46]. Thereafter, *Saccharomyces* species, principally *S. cerevisiae*, dominate and complete the fermentation process. *S. cerevisiae* performed aerobic respiration of sugars to obtain CO_2_, H_2_O, ATP and, consequently, pyruvic acid, glycerol and other biochemical intermediates. As sugar degradation progresses and the oxygen concentration decreases, *Saccharomyces* cells activate their anaerobic metabolism useful to transform sugars into alcohol, CO_2_, glycerol, acetaldehyde, acetic acid (responsible for volatile acidity), ethyl acetate and other alcohols that determine the taste qualities of the wine [47]. To prevent the risk of sluggish or stuck fermentations and quality deterioration, selected indigenous or commercially available *S. cerevisiae* strains with excellent enological characteristics are added as starter cultures to drive the transformation of the musts to wines of high quality and stability. 

During winemaking production, the fermentative activity of yeasts is influenced by the quality of the musts and by the presence of molecules produced by the activities of microorganisms associated with the berry, grape stalk, leaves and wood of the grape. *Botrytis cinerea* Pers. (teleomorph *Botryotinia fuckeliana* (de Bary) Whetzel), the causal agent of gray mold rots in wine and table grape vineyards, produce “botryticine” substances, heat-stable glycoproteins with molecular weights between 10 and 50,000 Da. These glycoproteins are accumulated in the berries and the must. Botryticine slows down fermentation kinetics and increases acetic acid and glycerol concentrations [48,49,50]. Microvinifications conducted with musts from vines affected by the Esca complex show qualitative differences compared to musts derived from noninfected vines [38,40].

In this study, the effects of the exopolysaccharide pullulan and two naphthalenone pentaketides, such as scytalone and isosclerone, on the growth and enological performances of two commercial strains of *S. cerevisiae* were evaluated in synthetic must. Byosal HS1 and IOC 18-2007, are universal and well-known commercial strains of *S. cerevisiae* suitable for the fermentation of all kinds of grape juice. These strains cope well under low pH and temperature in the range of 8–30 °C, utilize the sugar in the must without the production of undesirable compounds, such as acetaldehyde, and are resistant to ethanol and sulfur dioxide (https://www.enartis.com; https://ioc.eu.com, accessed on 29 July 2022). 

Esca complex is associated with extensive wood necrosis in the trunk and branches through the development of pathogenic fungi [1,2,3,4,5,6,7,8,9]. The cultivar, pruning methods, climate conditions, nutritional status and vine physiology interfere with fungal colonization of the wood, lead to symptom expression on leaves and berries and made it difficult to individualize the diseased plants in the vineyard [1,2,3,4,5,6,7,8,9,14,22,23,24,30,31,32,33]. 

Pullulan, scytalone and isosclerone are phytotoxic secondary metabolites produced by *P. minimum* and *P. chlamydospora* the two most diffuse etiological agents of brown wood streaking, Petri disease and GLSD [25,26,27,51]. These three metabolites are found in wood, bleeding xylem sap and leaves of vines with symptoms of brown streaking associated with the Esca complex fungal pathogens [14,28,51]. Rachis, skin and pulp of ‘Italia’ vines naturally infected with *P. chlamydospora*, and *P. minimum* accumulated pullulan, scytalone and isosclerone [14]. Therefore, if these metabolites are present in berries of wine grapes, could be potentially found in the grape juice and must. 

When bio-assayed, these secondary metabolites showed different degrees of phytotoxicity and are associated with fungal lifestyles [15,25,26,51]. Scytalone and isosclerone are also involved in the melanin biosynthesis pathway of *P. chlamydospora*, *P. minimum*, *Verticillium dahliae* Kleb., *Berkeleyomyces basicola* (Berk. & Broome) W.J. Nel, Z.W. de Beer, T.A. Duong & M.J. Wingf. (syn. *Thielaviopsis basicola* Berk. & Broome) Ferraris) and *Pyricularia oryzae* Cavara [26]. Pullulan is nontoxic, nonirritating, nonionic, blood compatible, biodegradable, nonmutagenic, nonimmunogenic and noncarcinogenic substance [52]. Moreover, pullulan can be used as an adhesive, it forms fibers and oxygen-impermeable films [53]. 

To avoid the presence of naturally compromise grape juice [39,40,48,50], including the presence of pullulan, scytalone and isosclerone in the berries, our experiments were conducted with synthetic must. Grape must composition moreover changes with the grape variety, ripeness stage, terroir characteristics, climate and viticultural factors [50]. In laboratory experiments, the use of natural grape must, to study yeast in enological variations, allows the best conditions [50]. However, data standardization and validation make it necessary to use synthetic must with defined composition [54,55].

Pullulan, scytalone and isosclerone are phytotoxins, or fungal secondary metabolites that exert toxicity against plants. Phytotoxins interfere with plant metabolisms, inhibit enzymatic activities, affect membrane integrity, disrupt mitochondrial functions, suppress defense responses and have an important role in the disease development. Some are phytotoxic only to the host plant susceptible to the pathogen (host-selective toxins); others exhibit general toxicities to both host and nonhost plants (non-host-selective toxins). Host- and non-host-selective toxins show distinct modes of action, target site and pathological role [15,56,57]. Phytotoxins also have unique and intriguing biological and chemical properties. For example, phytotoxic secondary metabolites produced by fungal plant pathogens have pharmaceutical potential as anticancer agents [58,59], potential use as agricultural pesticides, herbicides or antimicrobials [60].

Here we demonstrate toxic effects of these substances against other fungal species (*S. cerevisiae*).

The data obtained in the microvinifications conducted in this study shows that pullulan and the pentaketides mix (scytalone and isosclerone in a 9:1 *w*/*w* ratio) influence the growth and metabolism of the two tested *S. cerevisiae* strains. 

Byosal HS1 and IOC 18-2007, responded differently in the growth tests according to their metabolic activity. Overall, biomass formation was correlated with the duration of the lag phase and the exponential growth rate was correlated with the fermentation time that characterizes the considered strain. At the end of the experiments performed in the control flasks with synthetic must alone, Byosal HS1 showed a growth rate greater than 6.1% of strain IOC 18-2007. Regarding the presence of pullulan and the pentaketides mix in the media, no effects were recorded when evaluated at 1 and 5 µg mL^−1^. Assayed at 100 and 1000 µg mL^−1^ these substances inhibit, while at 10 µg mL^−1^ significantly reduce the growth rate both *S. cerevisiae* strains evaluated. 

Based on these data, the concentration of 10 µg mL^−1^ for both pullulan and pentaketides mix, was selected to perform microfermentations suitable to verify the effects on the enological performance of both *S. cerevisiae* strains.

At the end of the microvinification differences were recorded in the enological behaviour of the two strains assessed as a function of the media (control, with pullulan and with pentaketides mix) considered.

In control media, no differences were recorded in ethanol production between the two strains. The presence of pullulan reduced the ethanol production in both strains, while the scytalone–isosclerone mix significantly reduced ethanol production only with the Byosal HS1 strain. It is evident that sugar metabolism has not been directed towards the production of ethanol via fermentation but has been used in other pathways. With high glucose concentrations in the substrate (over 9 g·L^−1^), *S. cerevisiae* metabolizes sugars only by fermentation, as there is repression (Crabtree effect) in the synthesis of mitochondrial enzymes and consequent degeneration of these organelles or with high glycerol presence [48,61,62]. The data of the microfermentations conducted in this study allow us to hypothesize that pentaketides and pullulan have “shifted” the transformation of sugars in glycerol–pyruvic fermentation. From the glycerol–pyruvic pathway, *S. cerevisiae* regenerates NAD+, produces ethanol, glycerol (as a protector against high osmotic pressures), succinic acid, acetic acid, diacetyl, acetoin, 2,3-butanediol, ethanal (also called acetaldehyde), higher alcohols and two types of esters: the acetates of higher alcohols and the esters of fatty acids. The acetates of higher alcohols give off different odors, such as glue (ethyl acetate), banana (isoamyl acetate) or rose (phenyl ethanol acetate). The esters of fatty acids give off a fruity aroma [63,64]. Because of this shift, a reduction in ethanol yield was recorded in the microfermentations of strain Byosal HS1 conducted with the presence of pullulan and pentaketides mix. Less sensitive to these fungal secondary metabolites was the strain IOC 18-2007.

In all the microvinification, the initial pH was adjusted to 3.4 with NaOH [55]. At the end of fermentations, a reduction in the pH was recorded. In general, the pH of the wine is between 2.8 and 3.8, while in the fermentations conducted here it also reaches values of 2.4. Wine acidity conditions freshness, tartness and crisp taste. 

While pH measures the concentration of H+ ions and is a ripeness indicator and it influences the sensory properties of wine [65], total acidity considers all the acids in wine, both fixed organic and volatile acids and titratable acidity reflects the acidity assessed by titration with a strong base to a fixed pH of 8.2. The predominant acids that generally occur in the grape and are present in must and wine are tartaric and malic acid, while citric, acetic, succinic and lactic acids will be produced during fermentation [66].

Byosal HS1 and IOC 18-2007 grown in the presence of pullulan reduce the concentration of tartaric acid. *S. cerevisiae* is considered unable to use tartaric acid [46]. The decrease in this compound recorded in our experiments could be due to a chemical–physical effect associated with pullulan presence that could react with tartaric acid and lower its solubility [53]. Tartaric acid precipitates in all wines at the end of fermentation, when as potassium salt, it becomes insoluble in alcohol. This is not a problem for wine consumers. From the fermentation vats to the bottle decanting, filtration, clarification and stabilization steps bring wine stability and warrant the absence of precipitates [50]. In this study, the wines were analyzed at the end of fermentation and were not stabilized. Of modest importance is the reduction in tartaric acid noted for the two isolates in the control theses and in the presence of pentaketides mix which could be due to spontaneous precipitation.

Of particular interest is the reduction in malic acid recorded in microfermentations conducted in the presence of pullulan. Predominantly, the Gram-positive bacterial species *Oenococcus oeni* (Garvie) Dicks completes the malolactic fermentation by the conversion of malic acid into lactic acid, while this possibility is reduced in the malo-ethanolic pathway conducted by *S. cerevisiae* [67].

Lactic acid is a by-product of fermentation obtained by reducing pyruvic acid by the enzyme lactate dehydrogenase. In microvinifications with pullulan, both strains slowed down the production of this organic acid compared to the controls and the presence of pentaketides mix. This is probably associated with a reduction in the activity of the enzymes involved in this metabolic pathway. 

Acetic acid is the main component of volatile acidity. The concentration of this organic acid during alcoholic fermentation varied with yeast species and strains, the composition of the must and physical factors [68,69]. Acetic acid production by *S. cerevisiae* occurs during the fermentation of the first 50–100 g·L^−1^ of sugar, then decreases and increases slightly at the end of fermentation [70]. Anaerobiosis, low pH (<3.1) and high pH (>4.0), certain amino acid or vitamin deficiencies in the must, also favoring the production of acetic acid by *S. cerevisiae*. In this study, the two strains used produce different amounts of acetic acid. The minor concertation of this organic acid was recorded for the Byosal HS1 strain in the synthetic media containing pullulan. This exopolysaccharide may have reduced the metabolism that leads to the production of acetic acid, or vice versa intensified its degradation.

The fermentation vigour, the speed at which yeast starts the fermentation, is expressed as grams of CO_2_ produced during the first 2–3 days after fermentation [71]. Higher values of fermentation vigour indicate greater weight loss due to the amount of CO_2_ produced and released by the Müller trap. Compared to the controls, the presence of pullulan and pentaketides mix reduced fermentation vigour in both tested strains. Values of fermentation vigour recorded from other commercial and indigenous *S. cerevisiae* starter strains are in the range of 3.30–4.47 [72]. The presence of pullulan, and especially pentaketides mix, reduce the fermentation vigour, indicating a low adaptation ability to the growth conditions.

A separate discussion deserves the content in total polyphenols. In wine, especially red wine, phenolic compounds contribute to its sensory, antioxidant and pharmacological characteristics. Phenolic compounds are secondary metabolites present in grapes and wine that can be formed and transformed during the winemaking process. 

In microorganisms, the pentose phosphate pathway plays a key role in the synthesis of aromatic acids: crucial components for protein biosynthesis and precursors of phenolic compounds, vitamins and cofactors. *S. cerevisiae* utilizes DAHP synthase (EC 4.1.2.15) to synthesize the aromatic ring from glucose [66]. Under the control conditions, the strain IOC 18-2007 produces low polyphenols than Byosal HS1. Compared to control, strain Byosal HS1 shows a reduction of 41,8 and 44,4% when grown on synthetic must amended with pullulan and pentaketides mix, respectively. Regarding strain IOC 18-2007, the presence of pullulan reduced polyphenols concentration (about 5%), while the pentaketides mix induce a low (4.5%) increment. These variations could be related to 1) the production of enzymes that hydrolyze anthocyanins; 2) the release of polysaccharides capable of binding polyphenols; 3) the adsorption of anthocyanins on the yeast wall [73,74,75].

Finally, we would like to point out a drawback of this study in the light of the interpretation and generalization of the obtained results. Because of the small number of repetitions and the use of synthetic must alone, the results should be treated with caution. Further tests are needed to verify the universality of the obtained findings, with a wider range of *S. cerevisiae* indigenous or commercially available isolates, species of enological interest (e.g., species of *Torulopsis*, *Cryptococcus*, *Candida*, *Rhodotorula*, *Hanseniaspora*, *Kloeckera*, *Metschnikowia*, *Pichia* and other components of berry’s microbiome) and well characterized natural must. However, data collected in this study point out the attention on the possible enological effects associated with the presence of scytalone, isosclerone and pullulan. 

## 4. Materials and Methods

### 4.1. Strains

Two commercial strains of *S. cerevisiae* [Byosal HS1 (Esseco s.r.l.—Divisione Enartis San Martino, Trecate NO, Italy) and IOC 18-2007 (Institut OEnologique de Champagne, Epernay Cedex, France)] were selected on the bases of their well-known enological properties. Both strains produce high-quality wines, work at low pH, in the range of 8–30 °C, are resistant to sulfur dioxide and tolerate over 15% of alcohol (https://www.enartis.com; https://ioc.eu.com, accessed on 29 July 2022).

Byosal HS1 and IOC 18-were rehydrated and kept as stock cultures at 4 °C on Yeast Peptone Dextrose Agar (YPDA: 1% yeast extract, 2% peptone, 2% dextrose, 2% Technical agar N° 3 from Oxoid Part of Thermo Fisher Scientific—Microbiology, Hampshire, UK) for short-term storage. Fresh cultures of each yeast were grown in YPD broth (pH 6.5) at 25 ± 1 °C, for 24 h in orbital agitation (120 rpm). Cells were collected by centrifugation (Thermo Scientific SL8R centrifuge; Thermo Fisher Scientific Inc., Waltham, MA, USA) at 3500 g for 15 min, resuspended in YPD broth added with 25% (*v*/*v*) glycerol and stored at −80 °C before use.

Stock cultures of *P. chlamydospora* strain Pc25 and *P. minimum* strain Pm33, isolated from Esca-affected grapevines cv Sangiovese in Valenzano (Bari, Italy), were stored on Potato Dextrose Agar (PDA) slant tubes at 4 °C and routinely grown on PDA at 25 ± 1 °C in the dark.

### 4.2. Production, Extraction and Purification of Secondary Metabolites

Pc25 and Pm33 were grown in stationary liquid cultures in 1 L Roux flasks containing 150 mL Oxoid Czapek Dox Medium Modified (CzMM) with 0.1% Oxoid yeast extract and 0.1% Oxoid malt extract, pH 5.7 at 25 ± 1 °C for 28 days in the dark. At harvest, the mycelial mat was removed by filtration on Miracloth (Calbiochem, La Jolla, CA, USA) and the filtrate was centrifuged (Thermo Scientific SL8R centrifuge) at 8 000× *g*, 4 ± 1 °C for 20 min [25,27]. The mycelial mat was drained on filter paper, weighed to record fresh weight and dried at 70 ± 2 °C for 48 h to measure mycelial dry weight.

For pentaketides extractions, culture filtrates (3 liters per strain) were brought to pH 4 with 1N HCl and extracted 4 times with ethyl acetate (1.5 liters each). The combined organic extracts were dried on anhydrous sodium sulphate and evaporated under reduced pressure. Separation was performed on preparative TLC [25]. Each TLC band was scraped off, dissolved in methanol and analyzed with a Prominence Shimadzu liquid chromatograph (Shimadzu Corporation, Kyoto, Japan) equipped with DGU-20A online degassing unit, CBM-20A system controller, LC-20A solvent delivery unit, CTO-20A column oven, SPD-20A UV-VIS detector settled at 280 nm and a Waters (Milford, MA, USA) Nova-Pak C18 (150 × 3.9 mm i.d., 4 mm particle diameter) analytical column, following a previous procedure [14]. To avoid the loss of considerable amounts of scytalone and isosclerone, these two metabolites were tested together using the mixture in the ratio of 9:1 (*w*/*w*; scytalone/isosclerone) as a combination of Pm33 and Pc25 productions (scytalone 23.0 + 13.0 mg L^−1^ and isosclerone 1.5 + 2.5 mg L^−1^).

For pullulan extractions, culture filtrates (3 liters per strain) were treated with two volumes of cold absolute ethanol [27]. Each precipitate was filtered through Whatman GFC filters, dried, lyophilized, washed three times with 10 mL of cold methanol and weighed. HPLC analyses were performed on a Prominence Shimadzu liquid chromatograph equipped with a Waters differential refractometer (model 410), and a 1000 Å Nucleogel GFC (300 × 7.7 mm i.d., Macherey-Nagel, Düren, Germany) column [27].

### 4.3. Pullulan and Pentaketides Mix Interaction with S. Cerevisiae Strains

Fermentations were conducted by inoculating the selected species in synthetic grape juice medium according to the OIV-OENO Resolution 370-2012 [76] using analytical grade chemicals purchased from Sigma-Aldrich (a part of Merck Corporation, Burlington, MA, USA). D-glucose and D-fructose (115 g L^−1^ each) were used as carbon sources. The available nitrogen was provided by 100 mg L^−1^ (NH_4_)2HPO_4_ and by a mixture of 19 amino acids (89 mg L^−1^ aspartic acid, 126 mg L^−1^ glutamic acid, 26 mg L^−1^ alanine, 188 mg L^−1^ arginine, 39 mg L^−1^ asparagine, 126 mg L^−1^ proline, 39 mg L^−1^ phenylalanine, 14 mg L^−1^ glycine, 51 mg L^−1^ glutamine, 51 mg L^−1^ isoleucine, 39 mg L^−1^ histidine, 76 mg L^−1^ leucine, 63 mg L^−1^ lysine, 39 mg L^−1^ methionine, 101 mg L^−1^ serine, 6.00 mg L^−1^ tyrosine, 89 mg L^−1^ threonine, 51 mg L^−1^ valine, 26 mg L^−1^ tryptophan). Salts solution contained 1.14 g L^−1^ K_2_HPO_4_, 1.23 g L^−1^ MgSO_4_∙7H_2_O, 0.44 g L^−1^ CaCl_2_∙2H_2_O, 198.2 µg L^−1^ MnCl_2_∙4H_2_O, 135.5 µg L^−1^ ZnCl_2_, 13.6 µg L^−1^ CuCl_2_, 32.0 µg L^−1^ FeCl_2_, 5.7 µg L^−1^ H_3_BO_3_, 29.1 µg L^−1^ Co(NO)_3_∙6H_2_O, 24.2 µg L^−1^ NaMoO_4_∙2H_2_O, 10.8 µg L^−1^ KIO_3_. Organic acids (5.00 g L^−1^ potassium tartrate, 0.20 g L^−1^ citric acid, 3.00 g L^−1^ L-malic acid), vitamins (20 mg L^−1^ myo-inositol, 0.40 mg L^−1^ pyridoxine hydrochloride, 0.40 mg L^−1^ nicotinic acid, 0.20 mg L^−1^ calcium pantothenate, 0.10 mg L^−1^ thiamine hydrochloride, 0.04 mg L^−1^ p-aminobenzoic acid, 0.04 mg L^−1^ riboflavin, 0.03 mg L^−1^ biotin, 0.04 mg L^−1^ folic acid), 10 mg L^−1^ ergosterol and 0.5 mL L^−1^ tween 80 were also added. The pH was adjusted to 3.4 using NaOH [57]. Concentrated solutions of each compound were prepared, filtered through 0.22 µm nitrocellulose membranes (Millipore filter, type GSWP) and added in adequate amounts before *S. cerevisiae* inoculations.

#### 4.3.1. Effects on Growth

Pullulan and pentaketides mix (scytalone/isosclerone 9:1/*w*/*w*) solutions were tested at 1, 5, 10, 100 and 1000 µg mL^−1^ of synthetic must. Yeast inoculation was standardized at 4 × 104 cells mL^−1^ starting concentration. All fermentations were conducted in 500 mL Erlenmeyer flasks with a cotton cap at 20 ± 1 °C under 12 rpm of orbital shaking in a GFL 3020 shaker (Helago, Kladská, CZ) for 18 days. Yeast cultures grown on synthetic must without pullulan and pentaketides mix were used as control. 

Yeast growth and viability were determined every 3 days by counting colony-forming units (CFU) after decimal dilution with YPD broth on YPDA solid medium.

All experiments were performed at least two times, each with three replicates. 

#### 4.3.2. Effects on Enological Parameters

Based on the results obtained during the previous experiments, the concentration of 10 µg mL^−1^ for both pullulan and pentaketides mix was selected to perform new microfermentations suitable to verify the effects on the enological performance of both *S. cerevisiae* strains.

Microfermentations were achieved in 100 mL of synthetic must in 500 mL Erlenmeyer flasks using 1 × 10^6^ CFU mL^−1^ of each *S. cerevisiae* strain as starting concentration at 20 °C with 12 rpm orbital shaking for 18 days.

Sugars, tartaric, malic, lactic and acetic acid and a total polyphenol concentration at the end of fermentation, were ascertained with a Hyperlab wine analyzer (Stereoglass S.r.l. San Martino in Campo, Perugia, Italy).

The pH of the wine was measured using a Basic 20 pH-meter with a glass XS Foodtrode Electrode. 

The total titratable acidity was measured by titrating each wine sample against 0.1 M NaOH using Bromothymol blue as the indicator and expressed as g L^−1^ of tartaric acid.

The ethanol content was determined using a Gibertini Digital Distilling Unit (Gibertini Elettronica s.r.l., Novate Milanese, Milano, Italy) following the OIV-MA-AS312-01B method [77].

Ethanol yield was calculated as the ratio between ethanol concentration (g L^−1^) and sugar consumed (g L^−1^).

The fermentation vigour was investigated according to the OIV-OENO Resolution, 370-2012 [76] in 100 mL Erlenmeyer flasks sealed with a Müller trap, containing 100 mL of synthetic must alone or amended with pullulan or pentaketides mix at 10 µg mL^−1^. Before inoculation, yeasts were grown for 24 h in a YEPD medium to obtain the mother culture. Flasks, inoculated with 1 × 106 CFU mL^−1^, were incubated at 20°C, under 12 rpm orbital shaking, for 48 h. The fermentation vigour was expressed as grams of CO_2_ (calculated as 2.5 ×·Δ weight) evolved by 100 mL of substrate [71].

### 4.4. Statistical Analysis

For each analyzed parameter, the mean and the standard deviation (sd) were calculated. Data were preliminarily subjected to Shapiro–Wilk’s test and Levene’s test to verify the normality of the distribution and the homoscedasticity of variances, respectively. Subsequently, the variance analysis (ANOVA) with a 95% confidence level and Fisher’s least significant difference (LSD) were performed. All statistical tests were conducted using SAS software version 9.0 for Windows.

## 5. Conclusions

In the awareness that in field conditions the effective concentrations of pullulan and naphthalenone pentaketides (scytalone and isosclerone) used in this study, have reduced chances of being reached in the berries and must, it seems reasonable to consider the results commented here in the light of their possible enological effects. Negative is the reduction in total acidity that would bring undesirable effects on the quality of wines, especially white ones. A reduction in the vitality of the yeast population could affect the organoleptic characteristics of the wine. Positive is the reduction of the alcohol content, following the trends of the market that requires wines that are not excessively alcoholic. Pullulan could be applied at the end of fermentation to reduce the concentration of tartaric acid and tartaric instabilities and improve the appearance of the wine. Further data are needed to confirm and validate the enological performance in freshly pressed grape juice. It would be interesting to evaluate the effects of pullulan, scytalone and isosclerone on the yeasts present in the grapes (e.g., *Candida zemplinina* Sipiczki, *Candida stellata* (Kroemer & Krumbholz) S.A. Mey. & Yarrow, *Metschnikowia pulcherrima* Pitt & M.W. Mill., *Kloeckera apiculata* (Reess) Janke, *Pichia* spp.) to reduce the use of antimicrobial products such as sulfites and candidate, especially the pullulan, as a possible food additive for enological use.

## Figures and Tables

**Table 1:** Biomass, recorded as fresh and dry weight, final pH values and yield of secondary metabolites produced by *Phaeoacremonium minimum* (Pm33) and *Phaeomoniella chlamydospora* (Pc25) grown 28 days on Czapek modified medium at 25 ± 2 °C in the dark ^1^.

Parameters	Pm33	Pc25
Biomass fresh weight (mg L^−1^)	15.3 ± 0.12	37.5 ± 0.37
Biomass dry weight (mg L^−1^)	4.2 ± 0.02	6.8 ± 0.04
Final pH	3.9 ± 0.2	4.0 ± 0.2
Ethyl acetate extracts (mg L^−1^)	183.0 ± 12	191.0 ± 10
Scytalone (mg L^−1^)	23.0 ± 2	13.0 ± 1
Isosclerone (mg L^−1^)	1.5 ± 0.3	2.5 ± 0.6
Ethanol precipitate (g L^−1^)	6.5 ± 0.2	7.2 ± 0.5
Pullulan (mg L^−1^)	430.83 ± 92	982.4 ± 0.3

^1^ Values are the means of 2 experiments with 10 replicates ± sd.

**Table 2:** Enological performance of *Saccharomyces cerevisiae* strains Byosal HS1 and IOC 18-2007 grown on synthetic must (Con) and synthetic must amended with 10 µg mL^−1^ of pullulan (Pul) or pentaketides mix (scytalone/isosclerone; 9:1/*w*/*w*; P-Mx) ^1^.

Parameters	Byosal HS1			IOC 18-2007		
	Con	Pul	P-Mix	Con	Pul	P-Mix
pH	2.40 ± 0.1 ^a^	2.44 ± 0.1 ^a^	2.44 ± 0.1 ^a^	2.40 ± 0.1 ^a^	2.86 ± 0.1 ^b^	2.40 ± 0.1 ^a^
Residual sugar (g L ^−1^)	0.1 ± 0.01 ^a^	0.4 ± 0.01 ^a^	14.79 ± 0.04 ^b^	0.29 ± 0.02 ^a^	0.12 ± 0.01 ^a^	35.31 ± 0.05 ^c^
Utilized sugar (g L ^−1^)	229.93 ± 0.01 ^a^	229.61 ± 0.1 ^a^	215.21 ± 0.4 ^b^	229.71 ± 0.2 ^a^	229.92 ± 0.1 ^a^	194.69 ± 0.2 ^c^
Ethanol (%)	7.5 ± 0.1 ^a^	4.1 ± 0.1 ^c^	2.44 ± 0.1 ^d^	7.3 ± 0.1 ^a^	5.7 ± 0.1 ^b^	6.9 ± 0.1 ^a^
Ethanol yield ^2^	0.32 ± 0.02 ^a^	0.17 ± 0.02 ^c^	0.11 ± 0.02 ^c^	0.31 ± 0.02 ^a^	0.24 ± 0.02 ^a^	0.35 ± 0.02 ^a^
Titratable acidity (g L ^−1^)	10.27 ± 0.1 ^a^	6.6 ± 0.1 ^b^	9.82 ± 0.1 ^a^	10.05 ± 0.1 ^a^	7.5 ± 0.1 ^b^	9.75 ± 0.1 ^a^
Acetic acid (volatile acidity) (g L ^−1^)	0.51 ± 0.01 ^a^	0.25 ± 0.02 ^c^	0.53 ± 0.02 ^a^	0.43 ± 0.02 ^b^	0.40 ± 0.01 ^b^	0.45 ± 0.01 ^b^
Tartaric acid (g L ^−1^)	4.14 ± 0.07 ^a^	3.08 ± 0.02 ^b^	4.29 ± 0.03 ^a^	4.27 ± 0.03 ^a^	3.25 ± 0.01 ^b^	4.29 ± 0.08 ^a^
L-malic acid (g L ^−1^)	1.46 ± 0.07 ^a^	0.95 ± 0.02 ^c^	1.43 ± 0.01 ^a^	1.45 ± 0.02 ^a^	1.05 ± 0.02 ^b^	1.51 ± 0.03 ^a^
Lactic acid (g L ^−1^)	0.05 ± 0.02 ^a^	0.01 ± 0.001 ^b^	0.05 ± 0.001 ^a^	0.06 ± 0.001 ^a^	0.02 ± 0.001 ^b^	0.06 ± 0.001 ^a^
Total polyphenols (mg L ^−1^)	464.04 ± 5.2 ^a^	269.88 ± 3.1 ^b^	257.91 ± 3.6 ^d^	261.75 ± 3.2 ^b^	248.88 ± 3.5 ^c^	273.63 ± 3.7 ^b^
Fermentation vigour ^3^	3.17 ± 0.01 ^a^	0.35 ± 0.07 ^c^	0.01 ± 0.001 ^d^	3.16 ± 0.001 ^a^	1.35 ± 0.01 ^b^	0.09 ± 0.001 ^d^

^1^ Values are mean ± sd. Data with different superscript letters within each row are significantly different (Fisher’s Least Significant Difference test; *p* = 0.05). ^2^ Expressed as g of ethanol produced per g of sugar consumed. ^3^ Evaluated at day 3 of fermentation as g of CO_2_ evolved by 100 mL of the substrate.

## Data Availability

Not applicable.
